# Probe-Ultrasonicated Thyme Essential Oil Nanoemulsions: Physicochemical Characterization and Application in Chicken Burgers

**DOI:** 10.3390/foods15071154

**Published:** 2026-03-28

**Authors:** Tamires Soares Schug, Marcia Foster Mesko, Larissa Riberas Silveira Teixeira, Thiago Castanho Pereira, Erico Marlon Moraes Flores, Elessandra da Rosa Zavareze, Carla Rosane Barboza Mendonça, Mariano Michelon, Eliezer Avila Gandra

**Affiliations:** 1 Graduate Program in Food Science and Technology, Federal University of Pelotas (UFPel), Pelotas 96010-900, Brazil; tamiresschug@gmail.com (T.S.S.); marciamesko@yahoo.com.br (M.F.M.);; 2 Postgraduate Program in Nutrition and Food, Faculty of Nutrition, Federal University of Pelotas (UFPel), Pelotas 96010-610, Brazil; larissariberas@outlook.com (L.R.S.T.);; 3 Department of Chemistry, Federal University of Santa Maria (UFSM), Santa Maria 97105-900, Brazil; thiago.castanho@acad.ufsm.br; 4 School of Chemistry and Food, Federal University of Rio Grande (FURG), Rio Grande 96203-900, Brazil; michelonmariano@gmail.com

**Keywords:** colloidal stability, antimicrobial activity, natural preservatives, processed meat products, microbiological quality, ultrasound-assisted emulsification

## Abstract

The bioactive compounds in thyme essential oil (TEO) have been investigated as natural preservatives. However, their direct application in foods is limited by their poor water solubility and high volatility. In this context, nanoemulsions represent promising delivery systems for bioactive compounds due to their improved physicochemical stability and functional performance. This study aimed to develop and characterize TEO nanoemulsions prepared by ultrasound-assisted encapsulation using an ultrasonic probe and whey protein concentrate as a surfactant, with potential application in chicken burgers. Different sonication times (1, 3, 5, 7, and 10 min) were evaluated, and ultrasonication time was evaluated as the experimental variable. The formulation processed for 3 min presented the smallest hydrodynamic diameter (289 nm) and a homogeneous spherical morphology. The nanoemulsions showed low cytotoxicity, maintaining cell viability above 90% at all evaluated concentrations. In vitro antibacterial assays demonstrated activity against *Staphylococcus aureus* and antifungal effects against *Aspergillus* and *Penicillium* species. When applied to chicken burgers, the treatment containing 100 ppm of nanoencapsulated TEO contributed to reductions in *S. aureus* and mesophilic aerobic microorganism counts during 7 days of refrigerated storage. These findings indicate that TEO nanoemulsions present potential as natural antimicrobial systems for food preservation applications.

## 1. Introduction

Modern consumers, driven by greater access to information, are actively choosing healthier food options. In this context, an increase in the demand for products with natural formulations and free from artificial additives has been observed, giving rise to the concept of “clean label” products [1]. The use of traditional chemical preservatives in meat and meat products has been increasingly questioned due to the potential toxicological effects associated with long-term consumption, driving the food industry to seek natural preservation alternatives [2].

In the processing of meat products, sodium and potassium nitrite and nitrate salts are widely used as curing agents due to their roles in color development, microbial inhibition, and control of lipid oxidation. However, under gastric conditions or during cooking at high temperatures, nitrite may react with amines to form N-nitroso compounds, including nitrosamines, many of which have recognized carcinogenic potential [3]. In addition, epidemiological studies have associated a high consumption of processed meats with an increased risk of colon, colorectal, and rectal cancer, reinforcing concerns about health impacts related to the use of synthetic additives and by-products formed during processing [4].

Among natural substances with potential to replace synthetic preservatives, essential oils (Eos) stand out due to their broad bioactivity. Eos consist of complex mixtures of low-molecular-weight organic compounds, predominantly volatile, synthesized by plants and found in different plant structures such as the leaves, flowers, seeds, and stems [2]. Among them, thyme essential oil (TEO, *Thymus vulgaris* L.) has gained prominence due to the presence of phenolic compounds such as thymol and carvacrol, recognized for their strong antimicrobial activity against foodborne pathogens such as *Escherichia coli* and *Staphylococcus aureus* [5,6].

Despite their potential as food preservatives, the direct application of Eos in foods is limited due to their physicochemical instability, low solubility in aqueous media, and volatility [7]. In this sense, nanoencapsulation emerges as a promising strategy to protect and enhance the bioactivity of Eos, as well as to improve their dispersion and stability in food systems [8]. Oil-in-water (O/W) nanoemulsions are systems in which oil droplets, generally smaller than 200 nm, are dispersed in a continuous aqueous medium and stabilized by a surfactant agent [2]. These structures exhibit greater kinetic stability against coalescence, flocculation, and gravitational separation than conventional emulsions, due to the reduced droplet size and the predominance of Brownian motion. In addition, the high specific surface area and optical transparency favor greater interaction between bioactive compounds and microorganisms, enhancing their biological efficiency [9].

One of the most applied techniques for the preparation of nanoemulsions is ultrasound-assisted emulsification (UAE), using ultrasonic baths or probes [10]. Ultrasound (US) is characterized as a mechanical wave with a frequency in the range of 20 to 10,000 kHz. In the food industry, the main applications use low-frequency (20–100 kHz) and high-intensity (>1 W cm^−2^) ultrasound for process intensification [11]. The interaction of US waves with liquid media leads to the induction of successive cycles of rarefaction and compression, resulting in the formation, growth, and collapse of microbubbles generated by local pressure variations. Although US theory is extensively discussed in the literature [12,13], in general, when low-frequency US is used, bubble collapse produces microjets and shock waves that increase turbulence and shear stress in the liquid medium, contributing to the intensification of emulsification and encapsulation processes [14].

In this sense, the UAE process is based on the generation of microjets and intense shear forces at the oil–water interface, promoting fragmentation of the dispersed phase and the formation of nanometric droplets. These droplets can be stabilized by the addition of surfactants, which reduce interfacial tension and help minimize coalescence and phase separation phenomena [14,15]. Parameters such as power and sonication time are determinants of the final properties of the nanoemulsion, directly influencing size distribution, stability, and biological activity [16]. Previous studies demonstrate that reducing the droplet diameter in nanoemulsions is directly associated with increased antimicrobial efficiency, mainly due to the greater specific surface area, which enhances the contact between active compounds and microbial cell membranes [17,18].

In this context, the application of nanoemulsions, prepared by UAE and containing TEO, in meat products represents an innovative alternative for the development of foods with lower levels of synthetic additives and greater microbiological and oxidative stability. Products such as chicken burgers are suitable matrices for this type of application, as they present high moisture and lipid and protein contents, making them susceptible to microbial spoilage. Thus, the incorporation of natural nanoemulsions into these products may contribute to shelf-life extension and quality improvement without compromising food safety and sensory acceptance [2].

Whey proteins have been widely investigated as natural stabilizing agents in colloidal systems due to their high adsorption capacity at the oil–water interface, forming efficient interfacial layers that contribute to the physicochemical stability of nanoemulsions. Whey protein acts predominantly through electrostatic repulsion mechanisms and, to a lesser extent, steric repulsion, reducing droplet coalescence and favoring the formation of homogeneous and kinetically stable systems [19]. In addition, studies show that the application of US in whey protein-stabilized nanoemulsions can promote partial denaturation of these macromolecules, exposing hydrophobic groups that enhance interfacial anchoring, resulting in smaller droplet sizes and better particle distribution. However, ultrasonic processing intensity and time are critical factors, since excessive conditions may compromise system stability [20].

Therefore, this study aimed to develop and characterize nanoemulsions containing TEO, sunflower oil, and whey protein as a surfactant, obtained using an ultrasound probe with different sonication times (1, 3, 5, 7, and 10 min). In addition, the application of these nanoemulsions in chicken burgers was evaluated by investigating their microbiological properties in order to evaluate the potential use of these formulations as natural preservatives in processed meat products.

## 2. Materials and Methods

### 2.1. Materials

White TEO (*Thymus vulgaris* L.) was commercially acquired from Ferquima–Indústria e Comércio de Óleos Essenciais (São Paulo, Brazil), packaged in a sealed amber bottle containing 100 mL. Sunflower oil, 80% whey protein concentrate, and the other ingredients used in the preparation of the meat product were purchased from a commercial retailer in the city of Pelotas, Brazil.

### 2.2. Preparation of the Nanoemulsions

The methodology used was adapted from Yang et al. [21], with modifications in formulation composition and processing parameters to suit the objectives of the present study and to obtain stable food-grade nanoemulsions containing thyme essential oil. The formulation composition and processing parameters were selected based on previous literature and preliminary trials to ensure the formation of stable nanoemulsions. The nanoemulsions were formulated (100 mL) using an oil phase (10%, *w*/*w*), composed of TEO (5%, *w*/*w*) and sunflower oil (5%, *w*/*w*), and an aqueous phase (90%, *w*/*w*), consisting of Milli-Q water and whey protein (1%, *w*/*w*), used as a surfactant. Initially, the aqueous phase was prepared by homogenizing whey protein in Milli-Q water using a magnetic stirrer for 10 min. Subsequently, the oil phase was weighed (10 g) and added to the aqueous phase, forming a pre-emulsion. This mixture was subjected to homogenization in a 150 mL glass beaker using an Ultra-Turrax (IKA T18 Digital, IKA Works, Staufen, Germany, model T18D 532, frequency 50/60 Hz, power 500 W, 19 mm probe) at 20,000 rpm for 3 min.

Thereafter, the pre-emulsion was subjected to an ultrasonication process using an ultrasonic probe (Sonics & Materials, Newtown, CT, USA, model VCX 750, power 750 W, frequency 20 kHz, probe diameter 12.7 mm), at 40% amplitude, with different processing times to obtain distinct formulations: A (1 min), B (3 min), C (5 min), D (7 min), and E (10 min). Throughout the entire procedure, an ice bath was used to prevent sample overheating.

In this study, ultrasonication time was considered the main experimental variable, while the remaining processing parameters were kept constant to allow the evaluation of its specific effect on nanoemulsion formation.

#### 2.2.1. Particle Size Distribution, Polydispersity Index, and Zeta Potential of the Nanoemulsions

Prior to analysis, 20 µL of nanoemulsion was diluted in 10 mL of ultrapure water. An equipment of dynamic laser scattering (Zetasizer Nano ZS, Malvern, Worcestershire, UK) was used for the measurements ifsize distribution and zeta potential. For zeta potential analysis, 1 mL of the diluted emulsion was introduced into a disposable capillary cell (DTS1060, Malvern, Worcestershire, UK) with the aid of a syringe to avoid bubble formation. For the size distribution determination, 1 mL of the sample was introduced into a plastic disposable sizing cuvette (DTS0012, Malvern, Worcestershire, UK). The measurements were carried out at a controlled temperature of 25 °C. For the laser diffraction method, prior to analysis, 100 µL of nanoemulsion was diluted in 10 mL of ultrapure water. The diluted suspension was introduced into a liquid dispersion unit (Hydro 2000, Malvern, UK), connected to a size analyzer (Mastersizer 2000, Malvern, UK), using water as a dispersing medium. To maintain a uniform flow, the sample was initially agitated at 1500 rpm; the stirring rate was subsequently lowered to 500 rpm during the actual data collection.

#### 2.2.2. Morphology of the Nanoemulsions

To evaluate the morphology of the nanoemulsions by transmission electron microscopy (TEM) (JEOL JEM-1400, equipped with a 20 μm aperture at 100 kV), the methodology described by Souza et al. [22] was followed. Aliquots of 50 μL of the nanoemulsions were deposited onto 200 mesh copper grids coated with Formvar (EM Sciences, Hatfield, PA, USA) and subjected to negative staining with 50 μL of 2% (*w*/*v*) uranyl acetate for 10 min at room temperature (25 ± 3 °C). Subsequently, excess liquid was removed using Whatman filter paper. Images were then acquired and subsequently processed.

#### 2.2.3. Phase Separation Analysis of the Nanoemulsions

The stability of the nanoemulsions was evaluated through phase separation analysis. Samples were visually examined for macroscopic instability phenomena, including phase separation, creaming, sedimentation, or the presence of residual foam on the surface.

For the analysis, glass tubes were filled with the nanoemulsions and kept at rest. Macroscopic changes, such as the formation of a top layer, foam on the surface, or sediment at the bottom of the tubes, were visually observed and recorded through macroscopic photographs. Evaluations were performed on the day following nanoemulsion preparation and after 15 and 30 days of storage at 25 ± 2 °C.

#### 2.2.4. Colorimetric Analysis and pH of the Nanoemulsions

The color of the nanoemulsions was determined using a colorimeter (Minolta, model CR-300), operating in the three-dimensional chromatic coordinate system. The pH of the samples was measured using a pH meter (Digimed, model DM-20), according to the methodology described by AOAC [23]. All analyses were performed in triplicate, and the results are expressed as mean ± standard deviation.

#### 2.2.5. In Vitro Cytotoxicity Analysis of the Nanoemulsions

The cytotoxic effects of the nanoemulsions on cell viability were evaluated using the murine fibroblast cell line L929 after 72 h of exposure. The cell line was obtained from the American Type Culture Collection (ATCC, Rockville, MD, USA) and maintained in Dulbecco’s Modified Eagle Medium (DMEM) supplemented with 10% fetal bovine serum (FBS; Gibco, Grand Island, NY, USA). The cells were grown under standard conditions in a humidified incubator at 37 °C with 5% CO_2_. For the assay, the cells were seeded in 96-well plates at a density of 5 × 10^3^ cells/well. Subsequently, they were treated with each nanoemulsion sample at concentrations of 50, 200, and 400 µg/mL for 72 h. Cells exposed to DMSO at a final concentration of 0.4% served as the control.

Cell viability was assessed using a 3-(4,5-dimethylthiazol-2-yl)-2,5-diphenyltetrazolium bromide assay (MTT; Sigma-Aldrich, St. Louis, MO, USA), following the method described by Mosmann [24]. After treatment, the culture medium was removed and replaced with MTT solution, and the plates were incubated at 37 °C for 90 min. Absorbance was measured at 492 nm using a microplate reader (SpectraMax 190, Molecular Devices, San Jose, CA, USA). Cell viability is expressed as a percentage relative to the control, and it was calculated according to Equation (1):

(1) Cell Viability (%) = (OD_492_ of treated cells/OD_492_ of control cells) × 100.

#### 2.2.6. In Vitro Antibacterial Activity of the Nanoemulsions

The antibacterial activity of the nanoemulsions was evaluated against *Escherichia coli* ATCC 43895 and *Staphylococcus aureus* ATCC 10832 using two phenotypic methods: the agar diffusion and micro-atmosphere methods. For each bacterium, the inoculum was standardized to 1.5 × 10^8^ CFU mL^−1^ (0.5 McFarland).

For agar diffusion, assays were performed on Mueller–Hinton agar following CLSI M02 guidelines [25], with adaptations for the well diffusion method. Bacterial suspensions were spread using sterile swabs, and wells (~6 mm) were filled with 30 µL of the nanoemulsion. Plates were incubated at 37 °C for 24 h, and measurements of inhibition zones were recorded in centimeters and performed in triplicate.

The antibacterial activity of the nanoemulsions in the micro-atmosphere was evaluated using the technique proposed by Ghabraie et al. [26], with modifications. Aliquots of 0.1 mL of bacterial cell suspensions were inoculated onto the surface of Mueller–Hinton agar plates. On the lid of each plate, sterile paper discs were placed, onto which different volumes of the antimicrobial substance (100 and 50 µL) were added. The plates were immediately closed in an inverted position and incubated at 37 °C for 24 h. Antibacterial activity is expressed as the percentage reduction in colony-forming units (CFU) in treatments containing the antibacterial substance compared with a control containing sterile water. The percentage of inhibition (I) was calculated according to Equation (2):

(2) I (%) = (nc − nt/nc) × 100

where *n*c is the colony count of the control group, and *nt* is the colony count of the treated group. The results are expressed as mean ± standard deviation.

#### 2.2.7. In Vitro Antifungal Activity of the Nanoemulsions

Antifungal activity was determined as described by Souza et al. [22]. The nanoemulsions (5 μL/mL) were added directly to potato dextrose agar (PDA), which was poured into sterile Petri dishes. PDA without nanoemulsion was used as a positive control for fungal growth. All plates were inoculated with mycelium of *Penicillium* and *Aspergillus* fungi, previously cultivated for 5 days on PDA, using a mycelial plug (0.5 cm) excised from the margin of a fungal colony with a cork borer. The plates were sealed with Parafilm and incubated at 25 °C. All treatments were performed in triplicate.

After 5 days of incubation, the colony diameter was evaluated by measuring fungal growth using a digital caliper (Mtx^®^, Shanghai, China, model 316119). The percentage of mycelial growth inhibition (MGI) was calculated according to Equation (3):

(3) ICM (%) = (dc − dt/dc − 0.5) × 100

where *dc* (cm) is the mean colony diameter of the control group, and *dt* (cm) is the mean colony diameter of the treated group. The results are expressed as mean ± standard deviation.

### 2.3. Microbiological Evaluation of Chicken Burgers Containing Nanoemulsion

For the development of chicken burgers, 300 g of meat batter was prepared. The formulation consisted of 69.4% lean chicken meat, 12.2% vegetable fat, 12.2% ice, 3.7% textured soy protein, 1.2% salt, and 1.0% seasonings (dehydrated parsley, dehydrated onion, dehydrated garlic, and black pepper). All ingredients were previously kept under refrigeration (≤4 °C). The chicken meat was ground, after which the vegetable fat, ice, soy protein, salt, and seasonings were added and manually homogenized until the meat batter was fully formed. After obtaining the base mixture, the corresponding treatment was incorporated: sodium nitrate curing salt (positive control, PC) or the nanoemulsion selected for the respective test (T1, T2, or T3) as described below:

Positive control (PC): meat batter supplemented with 150 ppm sodium nitrite.

Treatment 1 (T50): meat batter containing 50 ppm TEO, added in the form of a nanoemulsion.

Treatment 2 (T100): meat batter containing 100 ppm TEO, added in the form of a nanoemulsion.

Treatment 3 (T150): meat batter containing 150 ppm TEO, added in the form of a nanoemulsion.

The mixture was manually homogenized again for 30–60 s to ensure uniform distribution of the ingredients. The batter was shaped into 3 burgers weighing approximately 90 g each, placed in Petri dishes lined with plastic film, and stored under refrigeration (4 ± 1 °C) until analyses were performed.

Microbiological analyses were selected based on the standards established by RDC No. 331/2019 [27] and IN No. 60/2019 [28] from ANVISA, which recommend the determination of *Salmonella* spp., *Escherichia coli*, and aerobic mesophilic bacteria counts for raw meat products. Additionally, *Staphylococcus aureus* analysis was included. The analytical methodologies followed official International Organization for Standardization (ISO) protocols for each microorganism [29,30,31,32].

Analyses were conducted on the chicken burgers on the day of production (day 0) and after 7 days of refrigerated storage, with the aim of evaluating the antimicrobial effectiveness of the selected nanoemulsion on the microbiological stability of the product over time. Microbial counts were obtained from at least three plates per sample, and the reported values correspond to the average counts.

### 2.4. Statistical Analysis

All experiments were performed at least in triplicate, and the results are expressed as mean ± standard deviation. Cytotoxicity assays were conducted in triplicate within three independent experiments. Data were analyzed by a two-way analysis of variance (ANOVA), followed by Tukey’s multiple comparison test. Statistical analyses were performed using GraphPad Prism version 9.5.0 (GraphPad Software, San Diego, CA, USA).

The effect of time on antibacterial and antifungal activities was evaluated separately for each microorganism using a one-way analysis of variance (ANOVA), followed by Tukey’s post hoc test. Differences were considered statistically significant at *p* < 0.05. These analyses were performed using Statistica software version 7.0 (StatSoft Inc., Tulsa, OK, USA).

## 3. Results

### 3.1. Particle Size Distribution, Polydispersity Index, and Zeta Potential of the Nanoemulsions

**Figure 1:**
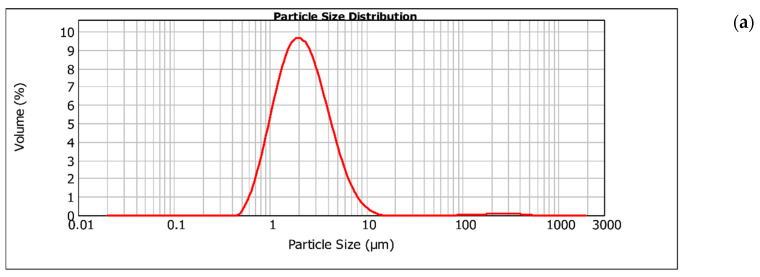

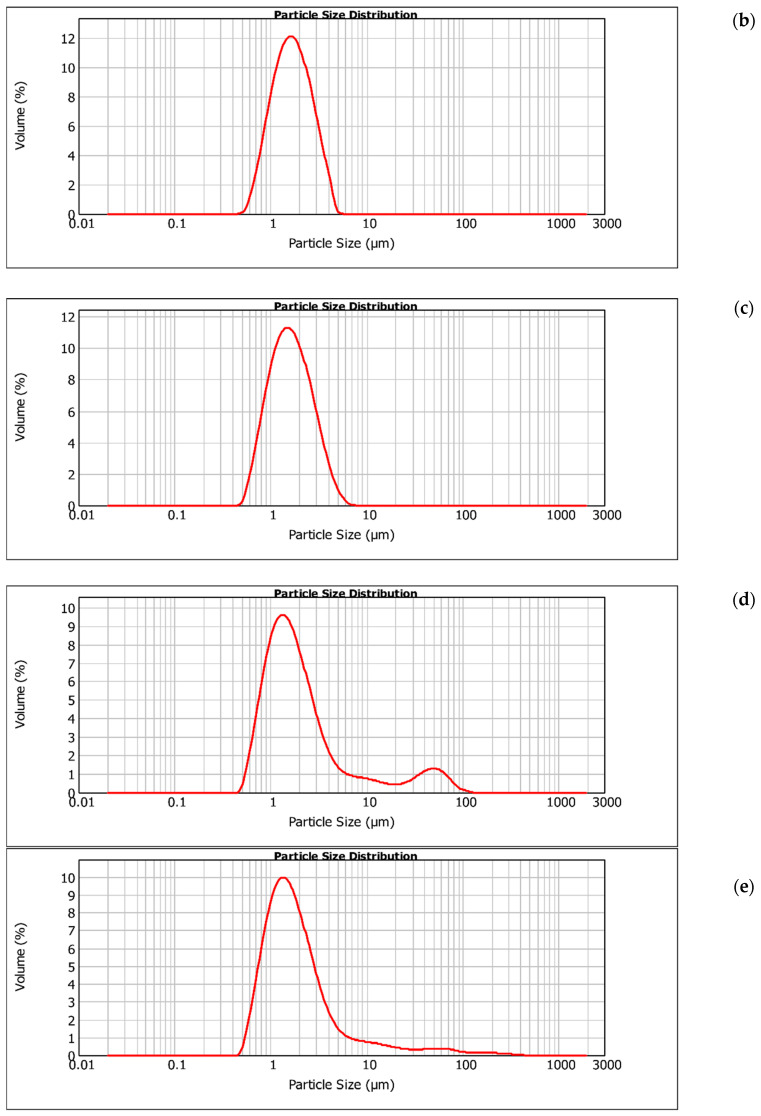
Volume-based particle size distribution of TEO nanoemulsions stabilized with whey protein produced at different sonication times: (**a**) 1 min, (**b**) 3 min, (**c**) 5 min, (**d**) 7 min, (**e**) 10 min, determined by laser diffraction.

The particle size distribution was evaluated by laser diffraction and dynamic light scattering to provide a comprehensive characterization of the dispersed system. Laser diffraction can provide a volume-based distribution, dominated by the particle population contributing the most to the dispersed-phase volume, in the micrometric range. On the other hand, dynamic light scattering can provide the intensity-weighted hydrodynamic diameter of the particles in the nanometric range [33]. Using the laser diffraction technique, it was found that the particle size distributions of the TEO nanoemulsions processed for 1 min (Figure 1a), 3 min (Figure 1b), and 5 min (Figure 1c) were predominantly narrow, with the highest volumetric fraction concentrated in the micrometric range, with a main peak approximately between 1 and 3 µm. According to SPAN values, particle distributions can be considered narrow (SPAN = 1.05–1.5), medium (SPAN = 1.5–4), and broad (SPAN = 4–10) [33]. The SPAN values for the samples processed for 1 min, 3 min, and 5 min were 1.819, 1.294, and 1.440, respectively. This classification was based on the low distribution width (SPAN), indicating a lower droplet size distribution and greater system homogeneity.

In contrast, the nanoemulsions subjected to longer sonication times exhibited broader distributions. The sample processed for 7 min (Figure 1d) displayed a multimodal profile, with the presence of a secondary population of larger particles, presenting a SPAN value of 7.561, suggesting greater structural heterogeneity. Similarly, the nanoemulsion processed for 10 min (Figure 1e) showed a wider distribution with an extended tail toward larger particle sizes and a SPAN of 3.282.

Dynamic light scattering analysis showed that all five evaluated formulations presented emulsion droplets in the nanometric range, with hydrodynamic diameters between approximately 289 and 448 nm (Table 1). The formulation processed for 3 min under ultrasonication exhibited the smallest mean droplet size (289 nm), which differed significantly from that of the other treatments (*p* ≤ 0.05). The polydispersity index (PDI) ranged from 0.420 to 0.702, in agreement with the results obtained by laser diffraction. Formulation A presented a significantly higher PDI value (*p* ≤ 0.05) than the other samples. The zeta potential values ranged from −24.83 to −37.97 mV, approaching the |30 mV| stability threshold [22]. Formulation B had the lowest zeta potential value, which differed significantly (*p* ≤ 0.05) from that of Formulations A, C, and E.

### 3.2. Morphology of Nanoemulsions

**Figure 2:**
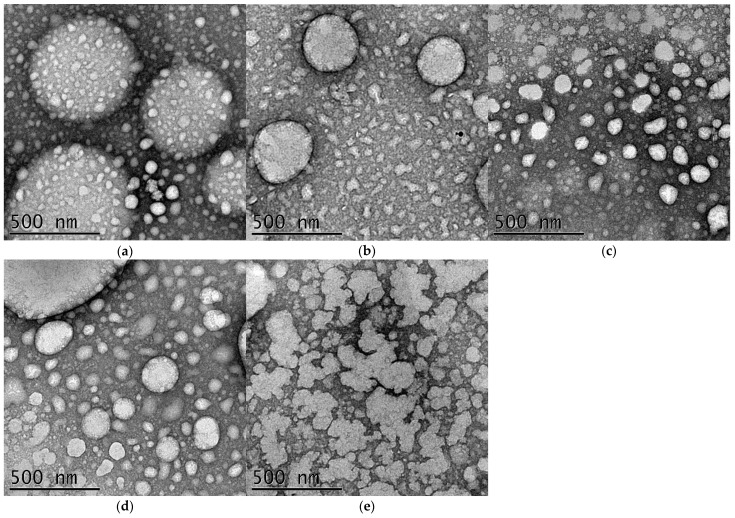
Transmission electron microscopy (TEM) images of TEO nanoemulsions stabilized with whey protein, produced by ultrasonication at different times: (**a**) 1 min; (**b**) 3 min; (**c**) 5 min; (**d**) 7 min; (**e**) 10 min. Scale bar: 500 nm.

TEM images (Figure 2) revealed differences in the morphology of the nanoemulsions as a function of ultrasonication time. Sample A (1 min) exhibited heterogeneous droplets, with the presence of larger structures and a wide size variation, indicating insufficient dispersion and heterogeneity in the particle distribution. Formulation B (3 min) showed more uniform and well-dispersed droplets. In Sample C (5 min), the formation of smaller, more spherical, and more homogeneous nanostructures was observed. Sample D (7 min) presented spheres of varying sizes. In contrast, Sample E (10 min) exhibited signs of structural instability, with aggregate formation and irregular shapes.

### 3.3. Phase Separation Analysis of the Nanoemulsions

**Figure 3:**
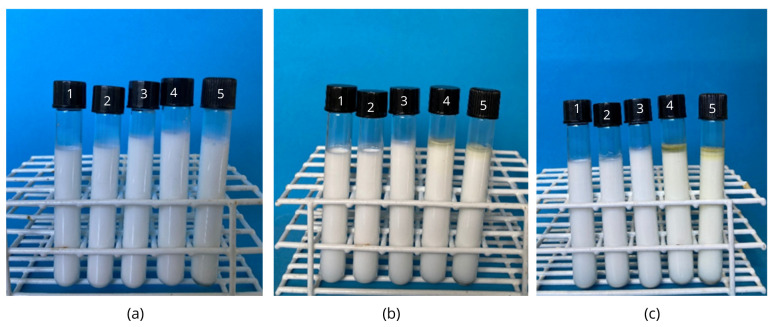
Macroscopic evaluation of the physical stability of TEO nanoemulsions stabilized with whey protein, produced by ultrasonication at different times: (1) 1 min, (2) 3 min, (3) 5 min, (4) 7 min, (5) 10 min, over 30 days of storage at 25 ± 2 °C. (**a**) Day 0; (**b**) day 15; (**c**) day 30.

The physical stability of the nanoemulsions was monitored over a 30-day period. On day 0, all nanoemulsion samples visually exhibited a homogeneous appearance, with no evidence of phase separation. The samples showed a uniform white coloration, with no surface foam formation or visible sedimentation (Figure 3a).

After 15 days of storage, physical stability was maintained in most samples (Figure 3b). Samples A, B, and C remained visually homogeneous, with no signs of creaming or sediment formation. Sample D showed a slight visual change, with the formation of a lighter upper layer, indicating the onset of phase separation. Sample E exhibited the most pronounced instability, with a more translucent upper layer and the possible onset of droplet coalescence.

After 30 days, the instability pattern observed in Samples D and E remained evident, whereas Samples A, B, and C remained stable throughout the storage period.

### 3.4. pH and Colorimetric Parameters of the Nanoemulsions

The pH values of the nanoemulsion samples ranged from 7.02 to 7.13. Sample A showed the lowest value (7.02 ± 0.05), whereas Samples C and D presented the highest values (7.13 ± 0.03). The remaining formulations exhibited similar values, with only minor variations (Table 2).

Regarding colorimetry, the L* parameter, which represents lightness on a scale from black (0) to white (100), ranged from 46.74 to 50.24, with Sample B showing the lowest luminosity and Sample C showing the highest. The a* values, which indicate color tendency along the green (negative values) to red (positive values) axis, were negative for all samples, ranging from −0.58 to −0.39, indicating a slight greenish hue, with minor differences among formulations. For the b* parameter, associated with the blue (negative values) to yellow (positive values) axis, values ranged from 2.76 to 2.94, indicating a mild yellowish tone across all nanoemulsions.

### 3.5. In Vitro Cytotoxicity of Nanoemulsions

**Figure 4:**
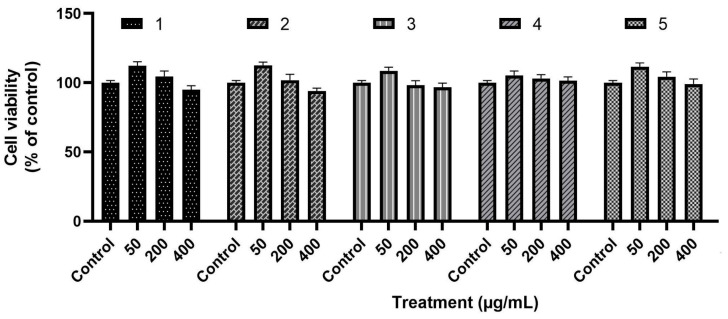
Cell viability (%) of the L929 cell line after 72 h of exposure to TEO nanoemulsions stabilized with whey protein, produced by ultrasonication at different times: (1) 1 min; (2) 3 min; (3) 5 min; (4) 7 min; (5) 10 min.

Figure 4 presents the cell viability (%) of the L929 cell line after 72 h of exposure to the TEO nanoemulsions at concentrations of 50, 200, and 400 µg/mL. All formulations maintained cell viability above 90%, indicating low cytotoxicity under the tested conditions. No statistically significant differences were observed among concentrations or formulations (*p* ≤ 0.05), suggesting that the evaluated nanoemulsions exhibit good biocompatibility after 72 h of exposure.

### 3.6. In Vitro Antibacterial Activity of the Nanoemulsions

**Figure 5:**
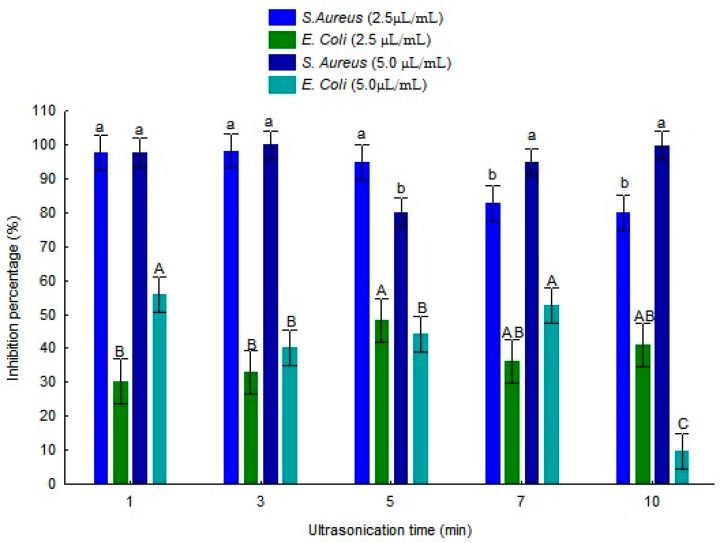
Percentage of inhibition, obtained using the micro-atmosphere method, of TEO nanoemulsions stabilized with whey protein, produced by ultrasonication at different times (1, 3, 5, 7, 10 min), against *S. aureus* and *E. coli* after 24 h. Different lowercase letters indicate statistically significant differences over time for *S. aureus*, while uppercase letters indicate differences for *E. coli*. Statistical analyses were performed independently for each concentration (2.5 and 5.0 µL/mL) using Tukey’s test (*p* ≤ 0.05).

The antibacterial activity of the nanoemulsions was evaluated using two approaches: the micro-atmosphere method and the well diffusion assay, both against *S. aureus* and *E. coli*. With the micro-atmosphere method (Figure 5), all formulations exhibited antibacterial activity against *S. aureus*. Formulation B stood out as the most effective, achieving 100% inhibition at a concentration of 5 µL/mL. Formulations A, D, and E also showed high inhibition levels, exceeding 94% at the highest tested concentration, whereas Sample C reached slightly lower values.

In contrast, the effect of the nanoemulsions on *E. coli* was limited. Overall, the inhibition percentages were lower than those observed for S. aureus, indicating greater resistance of the Gram-negative bacterium to TEO. At a concentration of 5 µL/mL, the highest inhibition percentages were observed for Formulations A and D, with values of approximately 55% and 52%, respectively. Formulation B showed intermediate inhibition, while Formulation E presented the lowest antibacterial effect. Despite the observed inhibition, none of the formulations achieved complete bacterial inhibition.

In the well diffusion assay (Table 3), only *S. aureus* exhibited detectable inhibition zones. Formulations B (16.3 ± 0.46 mm) and E (15.6 ± 0.28 mm) showed the largest halos, indicating greater diffusion of the active compounds and more pronounced antibacterial activity. The smallest inhibition zones were observed for Sample C (9.1 ± 0.25 mm). These results demonstrate that the TEO nanoemulsions stabilized with whey protein exhibited significant antibacterial activity against *S. aureus*, particularly Formulations B and E.

### 3.7. In Vitro Antifungal Activity of the Nanoemulsions

**Figure 6:**
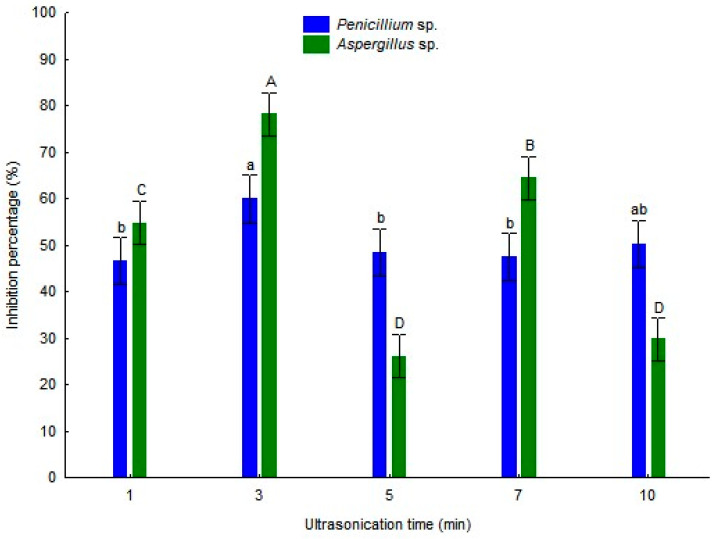
Percentage of fungal growth inhibition of TEO nanoemulsions stabilized with whey protein against *Aspergillus* sp. and *Penicillium* sp. Different lowercase letters indicate statistically significant differences over time for *Penicillium* sp., while uppercase letters indicate differences for *Aspergillus* sp. (Tukey’s test, *p* ≤ 0.05).

Figure 6 shows the percentage of growth inhibition of *Penicillium* and *Aspergillus* upon application of the TEO nanoemulsions incorporated into agar at a final concentration of 5 µg/mL. For *Penicillium*, Formulation B exhibited the highest antifungal potential, reaching 59.89% inhibition, which significantly differed (*p* ≤ 0.05) from that of the other samples, except for Sample D. Formulations A, C, and D showed moderate inhibition, ranging from 46.52% to 48.30%.

Regarding *Aspergillus*, Formulation B also presented the highest inhibition (75.67%), standing out from the other formulations. Formulations D and A showed intermediate activity, at 64.42% and 54.68%, respectively.

### 3.8. Microbiological Evaluation of Chicken Burgers Containing Nanoemulsion

For the application of nanoemulsions as preservatives in chicken burgers, Formulation B (with 3 min of processing) was selected based on its favorable performance in the antibacterial and antifungal analyses conducted in this study. Among the evaluated formulations, this nanoemulsion showed consistent antimicrobial activity across different assays, together with suitable physicochemical characteristics, including droplet size distribution and polydispersity index (PDI), which supported its selection for application in the food matrix.

Microbiological analysis of the chicken burgers was conducted to evaluate the presence of *Salmonella* spp., S. aureus, *E. coli*, and mesophilic aerobic bacteria after 0 and 7 days of refrigerated storage (Table 4). Microbial counts are expressed as average values obtained from at least three plates per sample. *Salmonella* spp. was not detected in any of the samples at either time point, indicating compliance with microbiological standards for this pathogen during the storage period evaluated. For *S. aureus*, on day 0, the counts ranged from 2.0 × 10^3^ to 1.1 × 10^6^ CFU/g, with treatment T100 presenting the lowest value and T150 presenting the highest. After 7 days of storage, the counts ranged from 4.5 × 10^2^ to 1.2 × 10^3^ CFU/g. These results may indicate a reduction in the population of this microorganism during storage under the evaluated conditions. The counts of *E. coli* on day 0 ranged from 3.0 to 11 MPN/g, while those on day 7 ranged from 3.0 to 15 MPN/g. These values suggest relatively stable populations during the storage period, without evidence of marked microbial growth. Regarding mesophilic aerobic bacteria, the counts on day 0 ranged from 2.7 × 10^4^ to 8.2 × 10^5^ CFU/g. After 7 days of storage, the values ranged from 2.4 × 10^3^ to 5.35 × 10^5^ CFU/g, which may indicate a tendency toward reduction or partial control of spoilage microbiota in some formulations.

## 4. Discussion

### 4.1. Particle Size Distribution and Polydispersity Index of the Nanoemulsions

The particle size analysis by laser diffraction showed that the nanoemulsions exhibited a predominantly unimodal distribution at the micrometric scale for ultrasonication times up to 5 min, with the highest volumetric fraction concentrated in the micrometric range. Longer ultrasonication times (7 and 10 min) resulted in broader, multimodal distributions, indicating that excessive ultrasonication may promote not only droplet disruption but also the recoalescence phenomenon and the formation of secondary particle populations, reflecting greater system heterogeneity. These effects can be explained by the physical action of US, including microjets and shear stress on the droplet surfaces. Regarding the results obtained by dynamic light scattering (DLS), the analysis showed mean diameters in the nanometric range, between 0.420 and 0.702, reflecting the fraction of smaller particles present in the system. This difference between methods was expected, as laser diffraction is more sensitive to larger particles and provides a volume-based distribution, whereas DLS emphasizes smaller particles through an intensity-based distribution [34]. Furthermore, the results suggest the presence of two particle populations at different size scales, encompassing micrometric and nanometric fractions. Therefore, the discrepancy observed between the distributions may be attributed not only to intrinsic differences between the analytical techniques but also to the characteristics of the emulsification process, which may have favored the simultaneous formation of larger primary droplets and a fraction of smaller nanostructures resulting from ultrasonic action. In summary, laser diffraction and dynamic light scattering analyses provided complementary data, with the former showing that the emulsions contained a micrometric fraction and the latter showing that they contained a nanometric fraction.

Al-Asmari et al. [35] reported that the preparation of Origanum and thyme EO nanoemulsions using a mixture of commercial surfactants (Tween 80 and Span^®^ 80) with a US probe (20 kHz, 550 W) resulted in a decrease in the droplet diameter as the ultrasonication time increased. This behavior highlights the influence of ultrasonication parameters and emulsifier systems on droplet size reduction during nanoemulsion formation. A reduction in droplet size further enhances the antimicrobial activity against bacterial membranes [35].

Doghish et al. [36] reported that thyme nanoemulsions prepared using Tween 80 as a surfactant and probe ultrasonication (350 W) for 20 min presented an average droplet size of approximately 91.28 nm and a PDI of 0.272. These values differ from those obtained in the present study, where the nanoemulsions showed larger particle sizes and higher PDI values while still maintaining physical stability, except for the formulations processed for 7 and 10 min, which exhibited phase separation. These differences may be associated with several factors, including the type and efficiency of the surfactant used, as well as the ultrasonication processing parameters. The reduction in droplet size in nanoemulsions is strongly influenced by the effectiveness and functionality of the surfactant at the oil–water interface, in addition to the operational conditions of the ultrasonication process.

The colloidal stability of nanoemulsions is closely associated with parameters such as droplet size, PDI, and zeta potential. In general, smaller droplets tend to exhibit greater kinetic stability due to intensified Brownian motion, which helps reduce sedimentation and coalescence phenomena [37]. Additionally, according to Stokes’ law, the creaming rate decreases proportionally to the square of the droplet diameter, contributing to improved physical stability of the system [19].

The uniformity of the system can be evaluated by the PDI, where values below 0.3 indicate a narrow size distribution, while values above 0.4 suggest a broader particle size distribution and a higher probability of coalescence [36]. In the present study, the PDI values ranged from 0.420 to 0.702, indicating a polydisperse system. Higher PDI values may suggest a greater tendency toward droplet aggregation and instability. This behavior was observed in Samples D and E, which showed phase separation during storage. In contrast, the other formulations remained visually stable during the evaluation period. These findings are consistent with those of the morphological analysis, which revealed heterogeneous droplet sizes and a polydisperse environment among the samples.

The zeta potential complements this evaluation, as higher absolute values contribute to greater electrostatic repulsion between droplets and, consequently, higher physical stability [19]. According to Souza et al. [22], values above |20| mV indicate adequate stability, whereas values above |30| mV are generally associated with highly stable systems when no additional steric stabilization is present. In the present study, the zeta potential values ranged from −24.83 to −37.96 mV, suggesting moderate-to-high electrostatic stability of the nanoemulsions. However, despite the relatively high zeta potential observed for Sample E (−37.96 mV), this formulation showed phase separation during storage. This behavior may be associated with the high polydispersity observed in the system, as indicated by the elevated PDI values, which reflect a heterogeneous droplet size distribution. Therefore, although electrostatic repulsion contributes to dispersion stability, other factors such as the droplet size distribution may also influence the physical stability of nanoemulsions.

Whey protein was used as a surfactant in the nanoemulsion formulations and may have contributed to system stabilization through electrostatic repulsion, as suggested by the negative zeta potential values obtained in this study. Serum proteins are known to adsorb at the oil–water interface, forming relatively thin interfacial layers that reduce interfacial tension, although such systems may remain sensitive to processing conditions [19]. In the present study, shorter ultrasonication times, as observed for Sample B, were associated with the formation of smaller and more uniform droplets. However, increasing the ultrasonication time may negatively affect stabilization due to the high energy generated by acoustic cavitation. Excessive exposure to ultrasound can promote partial protein denaturation, leading to interfacial rearrangements and possible protein aggregation. These structural changes may reduce the surface activity of the proteins and weaken the interfacial layer surrounding the droplets, thereby increasing the susceptibility of the system to coalescence and phase separation.

### 4.2. Morphology of the Nanoemulsions

The morphological analysis results directly support the stability findings, particularly for Samples D (7 min) and E (10 min), which exhibited droplets of varying sizes, an irregular distribution, and the absence of the spherical shape expected for stable nanoemulsions. This heterogeneity is consistent with the stability analysis images (Figure 3), where both formulations already showed phase separation after 15 days. Previous studies indicate that essential oil nanoemulsions generally exhibit a spherical shape and a homogeneous distribution when adequately stabilized [36,38]. The literature further emphasizes that ultrasonication time plays a crucial role in nanoemulsion formation and stability, with a sufficient processing time required to ensure a homogeneous droplet size and low PDI [37].

In this context, Doghish et al. [36] demonstrated that using an ultrasonic probe for 20 min (20 kHz, 350 W) produced a stable TEO nanoemulsion with a nonionic surfactant (Tween 80), which favored efficient droplet fragmentation. Meanwhile, Bleoanca et al. [18] obtained stable nanoemulsions using only 3 min of probe ultrasonication (20 kHz, 100 W 35% amplitude) with whey protein concentrate, highlighting the high efficiency of serum proteins in interfacial adsorption and steric stabilization of droplets. In addition, the authors worked with moderate emulsion volumes and optimized biopolymer concentrations, favoring the formation of kinetically stable systems in shorter processing times. These differences reinforce the notion that nanoemulsion stability depends not only on ultrasonication time but also on the type of equipment (probe or bath), frequency, applied power, sample volume, and, importantly, the nature of the surfactant or stabilizing biopolymer.

The instability observed in Samples D (7 min) and E (10 min) may be related to both the ultrasonication time and the system’s ability to prevent physicochemical processes such as coalescence, an irreversible phenomenon that promotes droplet growth. Prevention of this instability depends directly on the presence of adequate repulsive interactions, either electrostatic or steric, provided by the chosen surfactant [19,37].

Previous studies have reported that protein-based emulsifiers can contribute to improved morphological control and kinetic stability in emulsion systems [37]. In the present study, the results suggest that Samples A, B, and C exhibited more favorable characteristics, which may be associated with the combination of ultrasonication time and the emulsifier used in the formulation.

### 4.3. Phase Separation Analysis of the Nanoemulsions

The evaluation of physical stability showed that the formulations exhibited distinct behaviors during storage. Nanoemulsions A, B, and C remained stable throughout the 30-day period, with no evidence of creaming, flocculation, or phase separation, indicating that the ultrasonication conditions applied in these formulations were adequate to produce kinetically stable systems. In contrast, Samples D and E exhibited phase separation after 15 days of storage, suggesting reduced stability under longer ultrasonication processing times.

The differences observed among the formulations appear to be associated with the effects of ultrasonication intensity on the structural organization of whey proteins at the oil–water interface. Although Samples D and E presented relatively high absolute zeta potential values, their higher polydispersity indices and the TEM micrographs revealed a more heterogeneous droplet size distribution and the presence of aggregated structures. These findings suggest that excessive ultrasonication energy may promote structural modifications in whey proteins, such as partial denaturation or interfacial rearrangements, which can weaken the protective interfacial layer and facilitate droplet interactions, ultimately leading to destabilization phenomena such as flocculation and phase separation during storage.

An interesting observation arose when comparing Samples A and E. Sample A presented the highest polydispersity index, indicating a broader droplet size distribution, which is consistent with the TEM images showing the coexistence of larger and smaller droplets. Despite this heterogeneity, the system remained physically stable during the storage period. In contrast, Sample E exhibited a lower polydispersity index but showed clear evidence of droplet aggregation in the TEM micrographs, followed by phase separation during storage. This behavior suggests that droplet size heterogeneity alone does not necessarily determine the physical stability of nanoemulsions. Instead, the structural integrity and functionality of the protein interfacial layer appear to play a more decisive role in maintaining system stability. Therefore, these results highlight the importance of optimizing ultrasonication processing conditions in order to preserve the functional properties of whey proteins and ensure the long-term stability of nanoemulsion systems.

### 4.4. pH and Colorimetric Parameters of the Nanoemulsions

The pH values of the nanoemulsions ranged from 7.02 to 7.13, remaining close to neutrality across all formulations. Statistical analysis indicated that some formulations presented significant differences (*p* ≤ 0.05). However, the overall variation among the samples was relatively small. These results suggest that the different ultrasonication times had a limited influence on the acidity of the systems. This proximity among pH values was expected, since all nanoemulsions were prepared using the same aqueous-phase composition, oil, and surfactant, and ultrasonication time alone generally does not promote significant changes in system pH, as observed in previous works [39,40].

Regarding the colorimetric parameters, the L* values ranged from 46.74 to 50.24. Statistical analysis revealed significant differences among the formulations (*p* ≤ 0.05), indicating that ultrasonication time influenced the luminosity of the nanoemulsions. These differences may be associated with variations in the droplet dispersion and light scattering properties within the emulsified systems. In colloidal dispersions such as nanoemulsions, smaller and more uniformly distributed droplets tend to enhance light scattering, which directly affects the perceived brightness of the samples. The visual appearance of the nanoemulsions also supports these observations. As shown in Figure 3a, the samples exhibited a white and milky appearance, characteristic of oil-in-water emulsions. This optical behavior results from the intense scattering of light caused by the dispersion of oil droplets within the continuous aqueous phase. Such light scattering contributed to the relatively high L* values observed in the colorimetric analysis.

For the a* parameter, negative values were observed for all formulations (−0.58 to −0.39), indicating a slight greenish tendency in the samples. Although statistical differences were detected among some treatments (*p* ≤ 0.05), the variations remained relatively small, suggesting that ultrasonication time had a limited influence on this parameter. Similarly, the positive b* values (2.76 to 2.94) indicate a mild yellowish coloration in all nanoemulsions. Statistical analysis also indicated significant differences among some formulations (*p* ≤ 0.05). This coloration may be related to the presence of natural pigments and phenolic compounds in thyme essential oil, which contribute to the optical properties of the emulsified systems.

### 4.5. In Vitro Cytotoxicity of the Nanoemulsions

In the present study, all TEO nanoemulsions exhibited cell viability greater than 90% at concentrations of 50, 200, and 400 µg/mL, indicating low cytotoxicity within the tested concentration range and suggesting a favorable safety profile for topical applications. The colloidal stability of the nanoemulsions, associated with the small droplet size and the presence of a protein-based surfactant system, may contribute to minimizing aggressive interactions with the cell membrane. These findings are consistent with the dose-dependent behavior widely described by Bairagee et al. [41], who demonstrated that phenolic compounds, flavonoids, and anthocyanins tend to maintain high cell viability at moderate concentrations and only trigger cytotoxic effects at higher doses. This same study emphasized that the cellular response to bioactive compounds depends on both the concentration and chemical composition of the formulations, reinforcing that the absence of toxicity observed in our study aligns with the expected pattern for matrices rich in natural metabolites.

Similar results were also reported by Dudi et al. [42], where osthole nanoemulsions maintained viability above 90% even at the highest tested concentration (160 μg/mL), without significant signs of toxicity. As noted by Kayiran et al. [43], the maintenance of high viability across all concentrations evaluated here suggests that nanoencapsulation can reduce direct cellular exposure to more reactive compounds, mitigating the toxic potential of the essential oil. Therefore, our findings confirm that the thyme nanoemulsion exhibits minimal cytotoxicity and is suitable for conducting antimicrobial assays, ensuring that the expected antimicrobial effect is not confounded with potential cellular damage.

### 4.6. In Vitro Antibacterial Activity of the Nanoemulsions

The TEO nanoemulsions exhibited significant antibacterial activity, particularly against *S. aureus*. In the agar diffusion assay, only the formulations showed measurable inhibition halos against this bacterium, with Formulations B and E displaying the largest diameters (16.3 and 15.6 mm, respectively). Although Sample E exhibited lower physical stability during storage than Sample B, this result does not necessarily contradict its antibacterial effectiveness. Antibacterial activity in agar diffusion assays is mainly influenced by the release and diffusion of bioactive compounds from the oil phase, whereas physical stability is associated with the structural integrity of the nanoemulsion during storage. Therefore, even formulations with reduced long-term stability may still demonstrate strong antibacterial effects under the conditions of an antibacterial assay.

These findings indicate that the nanostructured formulations are particularly effective against Gram-positive microorganisms, which aligns with the expected behavior of hydrophobic compounds. As described by Al-Asmari et al. [35], hydrophobic agents can more easily penetrate the thick but porous cell wall of Gram-positive bacteria, while the outer double membrane of Gram-negative bacteria, such as *E. coli*, hinders penetration and provides greater resistance.

Higher sensitivity of *S. aureus* was also reported by Al-Asmari et al. [35], who observed inhibition zones of 18.32 mm for *S. aureus* and 10.32 mm for *E. coli*. Even more pronounced results were reported by Ozogul et al. [44], where US-produced thyme nanoemulsions showed 26.1 mm inhibition against *S. aureus*. This behavior may be related to the increased dispersion of the oil phase and the improved interaction between bioactive compounds and bacterial cells in nanoemulsion systems, as reported in previous studies.

The micro-atmosphere technique provided insights complementary to those of the diffusion assay. Using this method, the nanoemulsions demonstrated even more pronounced efficacy against *S. aureus*, particularly Formulation B (3 min), which achieved 100% inhibition at a concentration of 5 µL/mL. The effectiveness observed in the micro-atmosphere assays may be partly attributed to the action of volatile compounds from the TEO released into the air, promoting broad contact with the microorganisms. Previous studies have shown that volatile fractions of essential oils maintain potent antimicrobial activity against bacteria and fungi, even without direct liquid-phase contact with the cells [45,46]. Moreover, comparisons between liquid and vapor phases indicate that the gaseous phase can be particularly useful in preservation or surface decontamination applications without altering the food matrix [47]. Thus, the combination of nanostructuring with the natural volatility of essential oil components represents a promising strategy for microbial control in meat products and other processed foods.

### 4.7. In Vitro Antifungal Activity of the Nanoemulsion

The TEO nanoemulsions showed antifungal activity against both Penicillium and Aspergillus. According to Tukey’s test (*p* ≤ 0.05), Sample B presented the highest inhibition percentage for both fungi. Although some formulations exhibited differences in physical stability and droplet size, these parameters did not show a direct correlation with antifungal activity. This behavior is consistent with previous findings, which demonstrate that nanoemulsions containing thymol and carvacrol have a greater capacity to interact with fungal membranes due to their reduced particle size, thereby increasing cellular permeability and consequently inhibiting mycelial growth [22,36]. Similar results were reported by Souza et al. [22], who observed a marked increase in the antifungal efficacy of nanoencapsulated thyme oil against *Penicillium digitatum*, reinforcing that nanostructuring optimizes the delivery of active compounds to the site of action. Likewise, Doghish et al. [36] demonstrated that thyme oil nanoemulsions exhibit strong activity against *Aspergillus brasiliensis* and *A. fumigatus*.

The proposed mechanisms for antifungal activity include disruption of cell membrane integrity, increased permeability, leakage of intracellular contents, and destabilization of essential organelles [36]. The nanoemulsion used in this study facilitated the diffusion and transport of the hydrophobic components of the essential oil to the fungal membrane, intensifying these effects. Thus, our results corroborate those in the literature and reinforce the potential of thyme nanoemulsions as natural alternatives for fungal control in food applications.

### 4.8. Microbiological Evaluation of the Chicken Burger Treated with Nanoemulsion

The microbiological results of the chicken burgers showed behavior consistent with the antimicrobial activity observed in the in vitro assays. The differences observed in microbial counts on day 0 may be attributed to the natural variability of the raw meat matrix, since fresh meat products often present heterogeneous initial microbial loads even when processed under similar hygienic conditions.

Nanoemulsion B (3 min) showed a high inhibitory capacity against *Staphylococcus aureus* in the in vitro assays. A similar tendency was observed in the burger samples, particularly for treatment T100 (100 ppm), which presented the lowest *S. aureus* counts both on day 0 (2.0 × 10^3^ CFU/g) and after 7 days of storage (4.5 × 10^2^ CFU/g). These results may be related to the enhanced availability of phenolic compounds in nanoemulsified systems, which can facilitate interactions with bacterial cell membranes, a mechanism frequently reported for nanostructured essential oils. For *Escherichia coli*, the lower inhibition observed in the micro-atmosphere technique (30–48% at 2.5 µL/mL and 9–55% at 5 µL/mL) was also reflected in the burger samples, where bacterial populations appeared to be less affected during storage. This behavior is consistent with the higher structural resistance of Gram-negative bacteria to lipophilic compounds [35].

When comparing the nanoemulsion treatments with the positive control containing sodium nitrite (CP), it was found that nitrite showed lower microbial counts for *E. coli* and mesophilic aerobic microorganisms in several conditions, particularly during storage. This observation is consistent with the well-established antimicrobial activity of nitrite, which is associated with the formation of reactive nitrogen species. For *S. aureus*, however, the nanoemulsion treatments, especially T100, showed microbial counts within the same order of magnitude as those observed for the nitrite-treated samples. These results suggest that the nanoemulsion may contribute to the control of this microorganism under the evaluated conditions.

The evolution of the microbial counts between day 0 and day 7 also indicated a tendency toward a reduction in or the maintenance of *S. aureus* populations in samples containing the nanoemulsion. Overall, these findings suggest that the nanoemulsion exhibited antimicrobial potential, particularly against Gram-positive bacteria, and that its behavior in the food matrix followed a tendency similar to that observed in the in vitro assays. Overall, the results indicate that the thyme essential oil nanoemulsion showed antibacterial potential in the chicken burger matrix, particularly against Gram-positive bacteria, suggesting that nanoemulsion systems may represent a promising natural strategy for improving microbiological stability in meat products. Although further studies are required to fully elucidate the antimicrobial mechanisms and optimize formulation parameters, the present findings suggest that thyme essential oil nanoemulsions may contribute to the control of microbial populations in meat products.

## 5. Conclusions

The present study developed and characterized nanoemulsions that contained thyme essential oil (TEO), sunflower oil, and whey protein as a surfactant and were produced using an ultrasonic probe under different sonication times. Physicochemical analyses indicated that Formulation B (with 3 min of processing) exhibited favorable characteristics in terms of its particle size, homogeneous distribution, and colloidal stability. Under these conditions, the nanoemulsions presented a spherical morphology and no evidence of coalescence phenomena. In vitro antibacterial assays showed inhibitory activity against *Staphylococcus aureus* and detectable activity against *Escherichia coli*, particularly for formulations with more suitable physicochemical characteristics. When applied to chicken burgers, the nanoemulsion treatments contributed to the control of microbial populations during 7 days of refrigerated storage. Reductions in *S. aureus* and mesophilic microorganism counts were observed in the treated samples, while *Salmonella* remained absent throughout the storage period. Overall, the developed nanoemulsions exhibited suitable physicochemical characteristics, satisfactory stability, and antimicrobial potential both in laboratory assays and when applied to a meat matrix. These findings suggest that TEO-based nanoemulsions may represent a promising approach for the incorporation of natural antimicrobial compounds in food systems. However, further studies are required to optimize formulation parameters and evaluate their long-term performance in food preservation applications.

## Figures and Tables

**Table 1:** Zeta potential, mean hydrodynamic diameter (Z-average), and polydispersity index of TEO nanoemulsions stabilized with whey protein.

Sample	Ultrasonication Time (min)	Zeta Potential (mV)	Mean Diameter (nm)	Polydispersity Index
A	1	−32.80 ± 3.45 a	448 ± 11.6 a	0.702 ± 0.08 a
B	3	−24.83 ± 2.46 b	289 ± 4.67 c	0.420 ± 0.01 b
C	5	−32.87 ± 1.72 a	442 ± 12.57 a	0.528 ± 0.03 b
D	7	−29.10 ± 0.92 ab	349 ± 18.38 b	0.486 ± 0.00 b
E	10	−37.97 ± 3.95 a	383 ± 24.76 b	0.527 ± 0.01 b

Note: Different letters in the same column indicate statistically significant differences among samples (Tukey’s test, *p* ≤ 0.05).

**Table 2:** pH values and colorimetric parameters (L*, a*, b*) of TEO nanoemulsions stabilized with whey protein.

Sample	Sonication Time (min)	pH	Color		
			L*	a*	b*
A	1	7.02 ± 0.05 b	48.02 ± 0.17 d	−0.48 ± 0.01 b	2.76 ± 0.02 b
B	3	7.09 ± 0.02 ab	46.74 ± 0.08 e	−0.39 ± 0.01 a	2.94 ± 0.02 a
C	5	7.13 ± 0.03 a	50.24 ± 0.05 a	−0.47 ± 0.01 b	2.78 ± 0.01 b
D	7	7.13 ± 0.03 a	49.01 ± 0.01 c	−0.55 ± 0.01 c	2.84 ± 0.03 b
E	10	7.08 ± 0.03 ab	49.69 ± 0.02 b	−0.58 ± 0.03 c	2.77 ± 0.06 b

Note: Different letters in the same column indicate statistically significant differences among samples (Tukey’s test, *p* ≤ 0.05).

**Table 3:** Inhibition halo of TEO nanoemulsions stabilized with whey protein, produced by ultrasonication at different times, against *S. aureus* and *E. coli*.

Samples	Sonication Time (min)	Inhibition Halo (mm)	
		*S. aureus*	*E. coli*
A	1	10.1 ± 0.25 bc	NI
B	3	16.3 ± 0.46 a	NI
C	5	9.1 ± 0.25 c	NI
D	7	14.0 ± 0.37 ab	NI
E	10	15.6 ± 0.28 a	NI

Note: Different letters in the same column indicate statistically significant differences among samples (*p* ≤ 0.05). NI: No inhibition zone detected.

**Table 4:** Microbiological counts of chicken burgers subjected to different treatments with TEO nanoemulsion stabilized with whey protein.

Sample	*Salmonella*	*Staphylococcus aureus* (CFU/g)	*Escherichia coli* (MPN/g)	Mesophilic Aerobic (CFU/g)
Time 0 (0 Days)				
CP	Absent	9.0 × 10^4^	3.0	8.0 × 10^5^
T50	Absent	1.1 × 10^6^	11.0	5.8 × 10^5^
T100	Absent	2.0 × 10^3^	3.6	2.7 × 10^4^
T150	Absent	8.0 × 10^6^	11.0	8.2 × 10^5^
Time 7 (7 Days)				
CP	Absent	6.0 × 10^2^	3.0	2.4 × 10^3^
T50	Absent	1.2 ×10^3^	15.0	4.5 × 10^4^
T100	Absent	4.5 × 10^2^	15.0	9.5 × 10^4^
T150	Absent	1.1 × 10^3^	6.2	4.9 ×10^5^

CP—positive control, chicken burger with 150 ppm sodium nitrite; T50—treatment: chicken burger with 50 ppm nanoemulsion; T100—treatment: chicken burger with 100 ppm nanoemulsion; T150—treatment: chicken burger with 150 ppm nanoemulsion.

## Data Availability

The original contributions presented in this study are included in the article. Further inquiries can be directed to the corresponding authors.

## Abbreviations

The following abbreviations are used in this manuscript:

EOs	Essential oils
TEM	Transmission electron microscopy
TEO	Thyme essential oil
CFU	Colony-forming units
MPN	Most probable number
